# Following up children born preterm

**Published:** 2017

**Authors:** P Vijayalakshmi, Clare Gilbert

**Affiliations:** Chief, Paediatric Ophthalmology & Strabismus, Aravind Eye Care System, Madurai, Tamil Nadu, India.; Professor of International Eye Health and Co-director: International Centre for Eye Health, London School of Hygiene & Tropical Medicine, London, UK.

**Figure:**
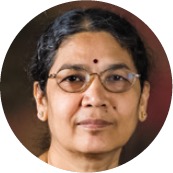
P Vijayalakshmi

**Figure:**
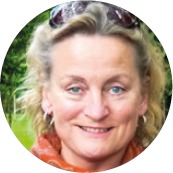
Clare Gilbert

**Babies born preterm, particularly those who have been treated for retinopathy of prematurity, are at greater risk of other eye conditions. Examining these children again, at the right time, can save their sight.**

**Figure:**
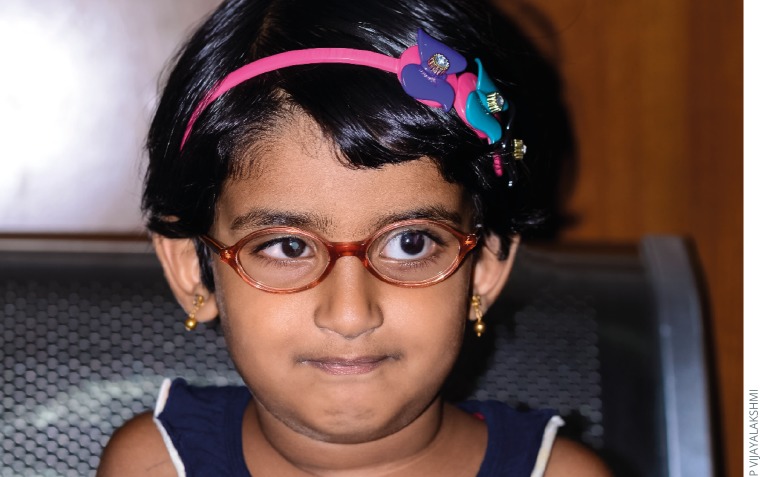
A six-year-old girl with stage 5 ROP in her right eye. Her left eye can see 6/60 after vitrectomy for stage 4b ROP and cataract surgery. INDIA

Preterm babies, and newborns who are unwell, are now surviving at higher rates globally than ever before. This is the result of expansion and improvement in services for sick and preterm babies. However, preterm birth is associated with a range of complications, including retinopathy of prematurity (ROP), and preterm infants are at a far higher risk of disabilities – including blindness – than healthy, full-term babies.^1^ Clinicians, together with low vision and rehabilitation specialists, can play a key role in reducing visual impairment and promoting normal development in this group of children.

The most common visual complications of prematurity are ROP and cerebral visual impairment (CVI), secondary to brain damage. CVI is associated with developmental delay and cerebral palsy. All preterm babies are at increased risk of refractive errors, particularly myopia, astigmatism, anisometropia (different refractive errors in each eye), and strabismus.^2^,^3^ All of these conditions increase with increasing prematurity. Some babies, particularly those who have been treated for ROP with laser, can develop cataract and glaucoma. The consequences of ROP can also lead to scarring and distortion of the retina, with loss of vision (Figure 1).

**Figure 1:**
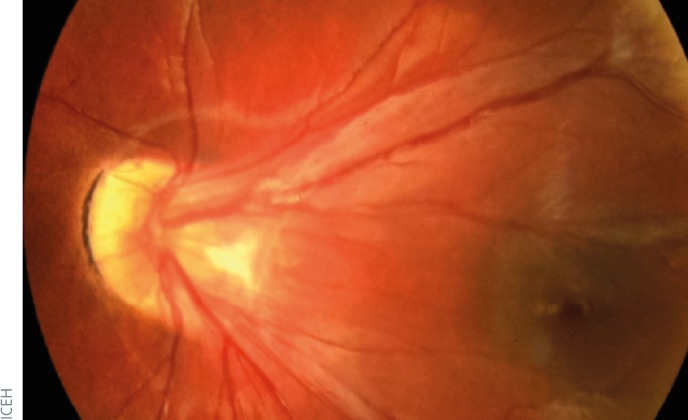
Scarring and distortion of the retina is one of the consequences of ROP.

## Refractive errors

In children who were born preterm, refractive errors have an early age of onset. It is important that any refractive errors are detected and managed properly in order to prevent amblyopia. However, it is important to bear in mind that the refractive status of the eyes changes dramatically over the first few years of life as the eyes grow, with most children's eyes becoming emmetropic (no refractive error) by the time they are 5–6 years old. It is thought that peripheral laser treatment for ROP, or the ROP itself, may interfere with these processes, leading to refractive errors.

### Myopia

Preterm babies are more likely to develop myopia than full term babies, even if they did not develop ROP. This is usually relatively low myopia, which develops at around the age of 4–5 years (the blue line in Figure 2). Babies who have developed any degree of ROP are at a higher risk than those who did not, and the myopia may be more severe and have an earlier onset (green line). Babies who have been treated for ROP using laser are at greatest risk, and may develop high myopia within a few months of treatment (orange line). Their myopia can progress rapidly before it stabilises (Figure 2).

**Figure 2:**
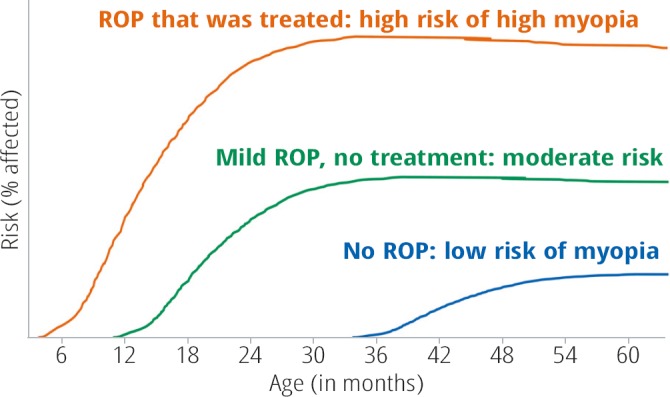
The risk and age of onset of myopia in children born preterm, depending on whether they developed ROP and whether they were treated for it

**Table 1:** Ocular complications of preterm birth and suggested timing of first examination

Preterm baby with …	What to look for	Level of risk	Timing of first and subsequent examinations
No ROP	Myopia	Low	At 2 years of age and annually thereafter
ROP, but no laser treatment needed	Myopia, astigmatism, strabismus	Moderate	At 1 year of age and annually thereafter
ROP, treated with laser	High myopia, astigmatism, strabismus, CVI, anisometropia	High	At 3 months of age and every 3–4 months to 2 years of age; then every 6 months to 6 years; and annually thereafter

### Astigmatism due to anisometropia

Astigmatism (due to an irregularly shaped cornea) and anisometropia are common, particularly following ROP treatment. Both can lead to amblyopia, which can be bilateral, if not detected and treated early. Treatment involves spectacle correction and daily intermittent occlusion of the better-seeing eye, with frequent follow-up visits.

### Strabismus

Strabismus (squint) is less common than refractive errors and may occur either in isolation or with a refractive error. Children with cerebral palsy following preterm birth are more likely to have strabismus. The degree of misalignment can vary over time, making the decision whether and when to operate more difficult than in children who were born at term.^3^

## Cerebral visual impairment and other eye conditions

Cerebral visual impairment should be suspected if the parents report that their child does not seem to see normally in the absence of any obvious ocular cause (although optic atrophy often accompanies CVI).

Cataract and glaucoma can develop either spontaneously or following treatment for ROP. The management of cataract and glaucoma in infants born preterm is extremely challenging, with glaucoma having a poor prognosis.

## Assessing and following up young children born preterm

It is recommended that all children who were born preterm are assessed by an ophthalmologist, particularly children who were treated for ROP and those with mild ROP which did not require treatment. However, there are no agreed guidelines for when this should be done. Table 1 gives some suggestions. At both initial and follow-up visits, consider the following:

- Is the child developing normally?
- Does the child seem to have normal vision?
- Is strabismus or nystagmus present?
- Does the retina look normal/healthy?
- Is there a significant refractive error?
- Are there any other eye problems, such as cataract?

Many parents believe that children born preterm develop more slowly than babies born at term. This is not the case in uncomplicated prematurity, and so it is important to assess the child's overall development (Table 2). Children born preterm are more likely to have global developmental delay (i.e., affecting all aspects of motor, social and cognitive development), or cerebral palsy, cognitive disability or autism. These children need to be identified early and referred for specialist care, for example to a developmental paediatrician or physiotherapist.

**Table 2:** Developmental milestones for children aged three months to 5 years

3 months	7 months	1 year	2 years	3 years	4 years	5 years
Begins to develop a social smile	Enjoys social play	Enjoys initiating play with others	Walks alone	Climbs well	Goes upstairs and downstairs without support	Swings, climbs, hops and somersaults
Raises head and chest when lying on the stomach	Transfers objects from hand to hand	Reaches sitting position without assistance	Points to objects or pictures when they are named	Turns book pages one at a time	Draws circles and squares	Says name and address
Watches faces intently	Ability to track moving objects improves	Bangs two objects together	Begins make-believe play	Uses 4 to 5 word sentences	Tells stories	Can count 10 or more objects
Smiles at the sound of your voice	Responds to own name	Responds to simple verbal requests	Demonstrates increasing independence	Sorts objects by shape and colour	Co-operates with other children	Likes to sing and dance
	Finds partially hidden objects					

**Figure 2:**
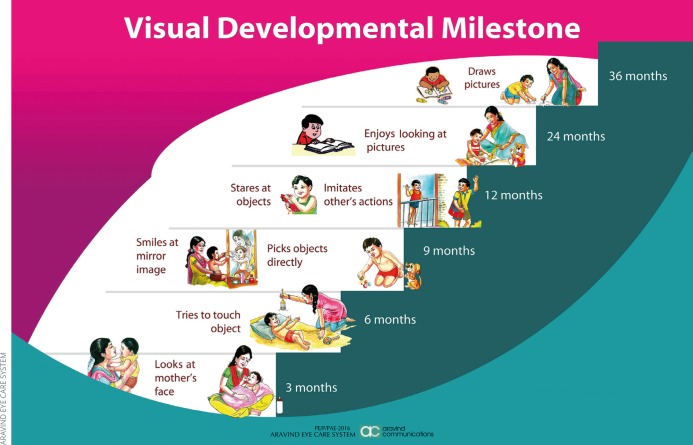
At every appointment, check whether children are achieving their expected visual milestones

Measuring visual acuity in young children is extremely difficult, but their visual functioning can be assessed using visual development milestones (Figure 3). Children who are irreversibly visually impaired or blind should be referred for vision rehabilitation.

Ocular alignment and eye movements should be assessed, and dilated examination of the retina and optic disc should be performed. Measure IOP and axial length when needed.

**NOTE:** Refraction should be performed with cycloplegia. If refraction is unreliable, consider refraction with atropine cycloplegia, under general anaesthesia.

## Prescribing and dispensing spectacles for young children

As a young child's visual world is near, it is not necessary to prescribe for, or fully correct, all simple myopia.

**Table 3:** Prescribing guidelines for young children born preterm

	3–18 months	18 months onwards
**Prescribe if …**	Sphere more than ±5D and/or Cylinder ≥2.5D and/or Anisometropia >1.5D	Sphere more than ±3D and/or Cylinder ≥2.5D and/or Anisometropia >1.5D
**Prescribe on an individual basis if …**	Sphere less than ±5D and Cylinder less than 2.5D and Anisometropia less than 1.5D	The refractive error is the same on two consecutive visits, two months apart

Suggestions for prescribing at different ages are shown in Table 3, which should be tailored to the individual child.

Young children do not have a well-formed bridge to their nose, and they require small frames and accurate centration of the lenses. The arms of the frame should fit around the ears, or the arms can be tied behind the child's head. Light, plastic lenses should be used.

## Counselling parents

Parents may be shocked and upset when they hear that their small child needs to wear spectacles or needs occlusion. This is particularly true for parents of babies who have been treated for ROP as they will already have had many anxieties and hurdles to overcome. Careful and repeated counselling is required to ensure that parents fully understand the need for their child to wear spectacles, that frequent follow-up will be required and the spectacles may need to be replaced.

## Summary

Children born preterm can have a range of complications which can impact on their development and the rest of their life. Successful management and the best possible outcome depends upon recognising and treating any problems as early as possible.

## References

[1] Beligere N Perumalswamy V Tandon M Mittal A Floora J Vijayakumar B Miller MT Retinopathy of prematurity and neurodevelopmental disabilities in premature infants. Semin Fetal Neonatal Med. 2015 Oct;20(5):346–53. 26235349 10.1016/j.siny.2015.06.004

[2] Fielder A et al. Impact of retinopathy of prematurity on ocular structures and visual functions. Arch Dis Child Fetal Neonatal Ed. 2014;0:F1–F6. 10.1136/archdischild-2014-306207 25336678

[3] Vijayalakshmi P Kara T Gilbert C Ocular Morbidity Associated With Retinopathy of Prematurity in Treated and Untreated Eyes: A Review of the Literature and Data From a Tertiary Eye-care Center in Southern India. Indian Peds 2016 53: Supplement 2:137–142. 27915322

